# Development of an inclusive 580K SNP array and its application for genomic selection and genome-wide association studies in rice

**DOI:** 10.3389/fpls.2022.1036177

**Published:** 2022-10-24

**Authors:** Kyu-Won Kim, Bhagwat Nawade, Jungrye Nam, Sang-Ho Chu, Jungmin Ha, Yong-Jin Park

**Affiliations:** ^1^ Center for Crop Breeding on Omics and Artificial Intelligence, Kongju National University, Yesan, South Korea; ^2^ Department of Plant Science, Gangneung-Wonju National University, Gangneung, South Korea; ^3^ Department of Plant Resources, College of Industrial Sciences, Kongju National University, Yesan, South Korea

**Keywords:** SNP array, genotyping, genome selection, genome assisted breeding, GWAS

## Abstract

Rice is a globally cultivated crop and is primarily a staple food source for more than half of the world’s population. Various single-nucleotide polymorphism (SNP) arrays have been developed and utilized as standard genotyping methods for rice breeding research. Considering the importance of SNP arrays with more inclusive genetic information for GWAS and genomic selection, we integrated SNPs from eight different data resources: resequencing data from the Korean World Rice Collection (KRICE) of 475 accessions, 3,000 rice genome project (3 K-RGP) data, 700 K high-density rice array, Affymetrix 44 K SNP array, QTARO, Reactome, and plastid and GMO information. The collected SNPs were filtered and selected based on the breeder’s interest, covering all key traits or research areas to develop an integrated array system representing inclusive genomic polymorphisms. A total of 581,006 high-quality SNPs were synthesized with an average distance of 200 bp between adjacent SNPs, generating a 580 K Axiom Rice Genotyping Chip (580 K _ KNU chip). Further validation of this array on 4,720 genotypes revealed robust and highly efficient genotyping. This has also been demonstrated in genome-wide association studies (GWAS) and genomic selection (GS) of three traits: clum length, heading date, and panicle length. Several SNPs significantly associated with cut-off, −log_10_ *p*-value >7.0, were detected in GWAS, and the GS predictabilities for the three traits were more than 0.5, in both rrBLUP and convolutional neural network (CNN) models. The Axiom 580 K Genotyping array will provide a cost-effective genotyping platform and accelerate rice GWAS and GS studies.

## 1 Introduction

Rice (*Oryza sativa*) is a staple food source for more than half of the global population (Wing et al., 2018). Rice production is expected to increase by 50–70% by 2050, with improved quality, reliability, and sustainability of global food demand (Zhao et al., 2011; Seck et al., 2012). However, sustainable production with fewer resources will require the efficient utilization of high-throughput and intensive systems in increasingly variable environments (Rasheed et al., 2017; Yu et al., 2022).

With advances in high-throughput sequencing technologies, -omics-based studies on rice have progressed considerably, enabling the efficient identification of a large number of single nucleotide polymorphisms (SNPs) (Wing et al., 2018; Nguyen et al., 2019). In addition to being highly prevalent, biallelic, codominant, and stable SNPs also play a significant role in phenotypic variation. SNPs are the most effective and highly informative genetic markers used to unravel functional variants underlying traits for crop improvement (Yu et al., 2022). Next-generation sequencing technologies enable accurate detection of SNPs from various genomic backgrounds. With the availability of several million SNPs, the challenge is efficient and economical genotyping of these SNPs (Rasheed et al., 2017).

High-throughput SNP genotyping is an attractive genotyping tool for identifying sequence polymorphisms (Rasheed et al., 2017; Guo et al., 2021). It is typically accomplished using SNP arrays or ‘chips’ or genotyping-by-sequencing (GBS). SNP arrays and GBS are cost-effective for genotyping thousands to millions of SNPs, whereas PCR-based genotyping requires hundreds to a few thousand SNPs, and is laborious, time-consuming, and suitable for small-scale genotyping. Although GBS has low ‘set-up’ and per-sample costs and performs SNP discovery and genotyping simultaneously, its experimental operation and data analysis are beyond the reach of average breeders (Verlouw et al., 2021). In contrast, high-throughput genotyping arrays can be used repeatedly to genotype different populations in a short period of time with straightforward data analysis (Rasheed et al., 2017).

Several genotyping platforms, including Illumina BeadXpress (Chen et al., 2011; Thomson et al., 2012), Fluidigm platform (Seo et al., 2020), Illumina Infinium (Yu et al., 2014; Thomson et al., 2017; Morales et al., 2020), and Affymetrix (Zhao et al., 2011; Singh et al., 2015; McCouch et al., 2016) have been developed and utilized in rice molecular breeding. RiceSNP50 was designed based on over 10M SNP loci from the resequencing data of 801 rice varieties (Chen et al., 2014). OsSNPnks include 50 K high-quality non-redundant SNPs (Singh et al., 2015). Because they mainly consist of SNPs within single-copy genes, SNP information has been widely applied in evolutionary and domestication-related studies of the *Oryza* genus. McCouch et al. constructed a high-density rice array consisting of 700 K SNPs surpassing the largest publicly available genotyping platform for any crop species (McCouch et al., 2016). Cornell_ 6 K _Array_Infinium_Rice (C6AIR) was designed and developed to be polymorphic within and between target germplasm groups and to map populations of interest (Thomson et al., 2017). Therefore, C6AIR provides a highly informative dataset to Cornell University and the IRRI, indicating the importance of data resources in designing SNP arrays. C6AIR was updated to C7AIR, covering polymorphisms between and within *O. sativa*, *O. glaberrima*, *O. rufipogon*, and *O. nivara* (Morales et al., 2020). Seo et al. developed two 96-plex indica-japonica SNP genotyping assays for particular target populations containing functional SNPs associated with agronomic traits for efficient genotyping (Seo et al., 2020). High-throughput genotyping platforms play critical roles in genetic diversity, gene mapping, germplasm resource analysis, genome-wide association study (GWAS), evolution analysis, and genomic selection (Xu et al., 2021).

**Figure 1:**
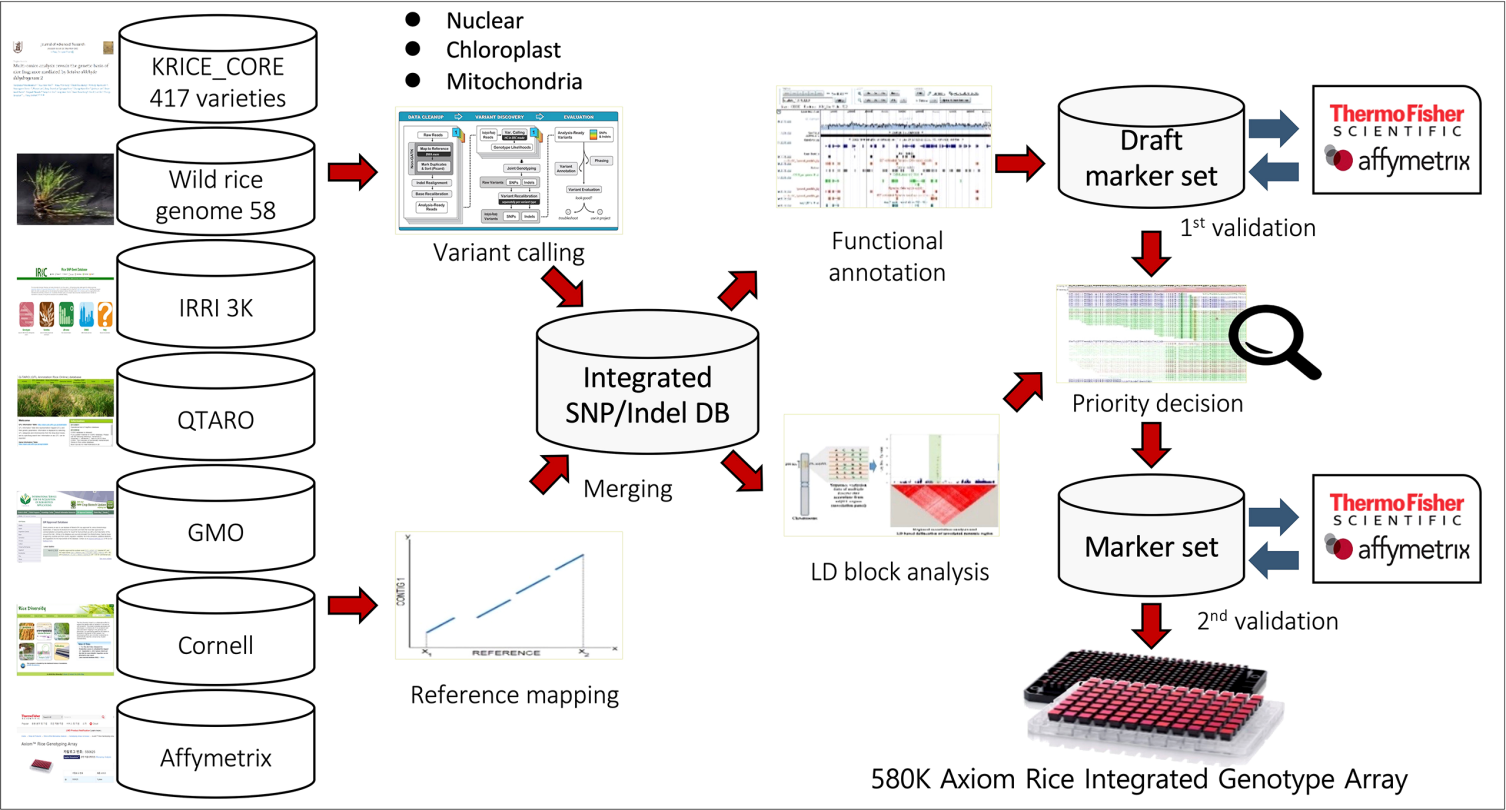
Schematic of 580K _ KNU chip design. SNPs were collected from eight different datasets, including 1) Resequencing data of 475 KRICE, 2) 3K-RGP from IRRI, 3) 700K rice array from Cornell University, 4) Affymetrix 44K Rice Chip, 5) QTARO database, 6) Plant Reactome Gramene database, 7) Chloroplast and mitochondria genomic sequence and 8) GMO information.

However, most of these arrays included whole-genome random SNPs but were not inclusive of SNPs related to key traits or research interests. Taking advantage of the accrued rice genomic sequence data, we collected SNPs from eight different highly informative datasets and selected high-throughput SNPs across the breeder’s research interests (**Figure 1**) to develop a large-scale genotyping array on the Affymetrix platform. Further validation of this array using a large set of accessions for GWAS analysis and genomic selection for different traits has demonstrated its usefulness in the global rice community.

## 2 Materials and methods

### 2.1 Sequence resources

We utilized different databases for designing the Axiom rice genotyping chip:1) Resequencing data of Korean World Rice Collection (KRICE) of 475 accessions composed of 417 cultivated and 58 wild accessions (Phitaktansakul et al., 2021), 2) Rice genome project data of 3,000 accessions (3 K-RGP) from the International Rice Research Institute (IRRI) (https://snp-seek.irri.org), 3) High-Density Rice Array (HDRA, 700K) array reported from Cornell university (McCouch et al., 2016), 4) Affymetrix 44 K Rice Chip (Affy44K) (Zhao et al., 2011), 5) QTARO database (http://qtaro.abr.affrc.go.jp/) (Yonemaru et al., 2010), 6) Plant Reactome Gramene Pathways database (https://plantreactome.gramene.org/) 7) Chloroplast (Tong et al., 2016; Cheng et al., 2019) and mitochondria (Tong et al., 2017) genomic sequences of japonica and rufipogon and 8) GMO, we used transgenic plants and genomes of various microorganisms as references for GMO marker design. The known GMO events consisted of host and insert regions; we utilized NCBI Primer-BLAST tool to get the full-length products and modified them into Axiom probes for 155 events. Besides, GMO markers were also developed from ds, tDNA, and tos17 as these insertion elements have been employed for generating large-scale mutant pools in different crops. We performed BLAST queries against rice reference genome sequences and unmapped sequences were selected for GMO marker design. Further, selected SNPs were aligned with different reference genomes including, japonica (ftp://ftp.ensemblgenomes.org/pub/plants/release-36/fasta/oryza_sativa/dna) (Kawahara et al., 2013), indica (http://rice.hzau.edu.cn/rice/download_ext/MH63RS2.LNNK00000000.fasta.gz) (Zhang et al., 2016), and *O. rufipogon* (https://www.ncbi.nlm.nih.gov/nuccore/NC_013816.1?report=fasta) (Fujii et al., 2010).

### 2.2 SNP filtering and integration

A VCF file was created from 3,475 rice accessions (3 K-RGP and KRICE), and SNP/indel sites with MAF<0.05, and missing rate> 0.1 were removed. Obtained SNPs were further enriched with Affymetrix (Affy44K), High-Density Rice Array (HDRA, 700K), and SNPs of selected genes from QTARO and Reactome databases, which resulted in a total of 7,682,442 markers. These markers were categorized into different classes viz., japonica, indica, and rufipogon specific, based on their sources (**Table S1**). The selected SNPs (called tag-SNPs) and the corresponding flanking sequences were submitted to Affymetrix (Axiom^®^ BioFx Services) service for initial probe screening. The priority was given as 0, 1, and 2, with 2 being the highest priority and 0 being the lowest. We assigned priority 2 to agronomically important genes, that have known pathways, and exist exclusively in either japonica or indica. After removing the tag-SNPs with a design score (pconvert)<0.6, a total of 3,204,347 SNPs met the Affymetrix probe designing criteria (**Table S1**).

### 2.3 Selection of SNPs for array development

**Figure 2:**
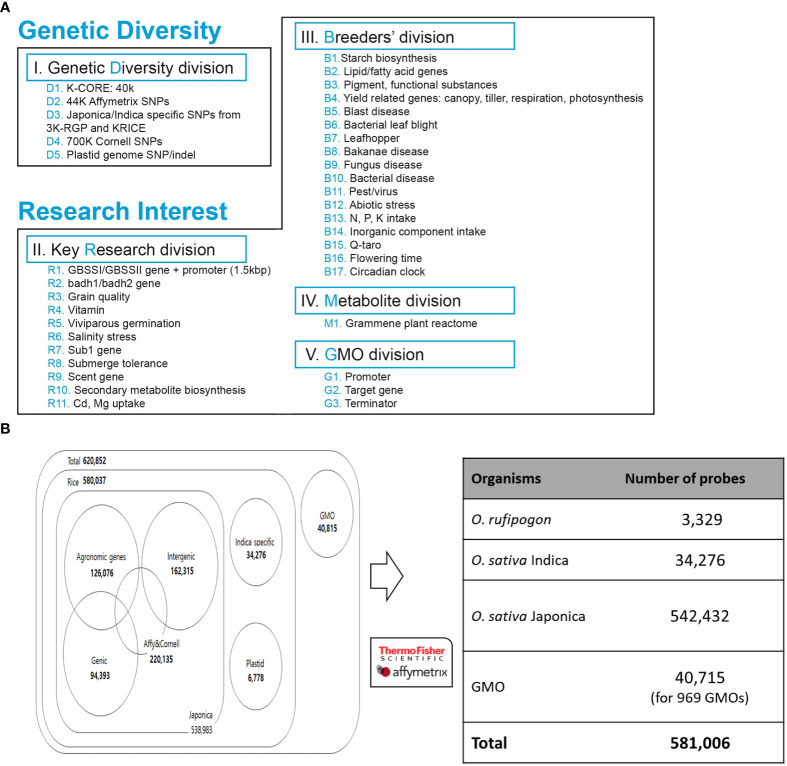
Development of integrated array system. **(A)** SNPs collected from genomic databases were prioritized based on breeders’ interest and key research divisions. **(B)** Summary of distribution of SNPs selected for Axiom 580K Array.

Among technical suitable variants, we allocated SNPs based on genetic diversity and breeder’s interest, which were divided into five divisions (**Figure 2A**). In the genetic diversity division, we selected 40 K high-quality SNPs from KRICE (Minimum allele frequency, MAF< 0.01, non-missing) (Phitaktansakul et al., 2021), 220,135 SNPs from the Affymetrix and Cornell chip, and SNPs specific to the indica/japonica group from the 3 K-RGP and KRICE data. We also selected chloroplasts (Tong et al., 2016; Cheng et al., 2019) and mitochondrial genomes (Tong et al., 2017) and filtered high-quality SNPs/indels (**Figure 2A**).

Furthermore, in the key research division, SNPs detected from 11 different genome-wide association studies (GWAS) for candidate genes were filtered with MAF< 0.01 (**Figure 2**). The evaluation of these studies resulted in 400 K polymorphisms, including 300 K SNPs and 100 K indels, which are associated with different traits.

In the breeder division, the key traits and genes of rice breeder interest were chosen, including starch synthesis, blast resistance, and yield-related genes (**Figure 2A**). A total of 1800 genes/QTL regions from the QTARO database were used for the SNP collection. In addition, for metabolite division, a set of genes was selected from the plant reactome (https://plantreactome.gramene.org) to retrieve SNPs involved in different pathways or processes in rice (**Figure 2A**, **Table S2**).

To prioritize probe sets for polymorphisms and select the final set of SNPs for array design, we used following criteria: (1) 71mer sequence (35 bp flanking sequence of target SNP) was to score the marker; (2) A marker was “not recommended” having one or more polymorphisms within 24 bases; (3) If a marker has the same recommendation for each strand, we recommended tilling the one with the highest pconvert value; (4) A marker/strand was recommended if: pconvert > 0.6, there are no wobbles, and poly count = 0; (5) A marker/strand was not_recommended if: duplicate count > 0, or poly count > 0, or pconvert< 0.4, or wobble distance< 21, or wobble count >= 3; (4) A marker was considered not_possible on a given strand if we cannot build a probe to interrogate the SNP in that direction.

A list of 620,852 candidate SNP loci was sent to Affymetrix Bioinformatics Services (Santa Clara, CA, USA) for array design. The quality of each SNP was assessed again and designated as ‘recommended’, ‘neutral’, ‘not recommended’, and ‘not_possible’ using *in silico* validation with proprietary software. We retained one SNP marker every 200 bp to ensure a uniform distribution and high density of SNPs throughout the rice genome. The final 580 K Axiom Rice Genotyping Chip (580 K _ KNU chip) contained a total of 581,006 SNP markers (**Figure 2B**).

### 2.4 Plant material and phenotyping

A total of 4,720 genotypes were genotyped for initial validation of the 580 K _KNU chip. This 4,720 genotype set was composed of different genotypes, including RILs (1,821), backcross inbred lines (209), backcross lines (BC1F3, 96), F_1_-F_7_ (209), breed (1,123), landraces (252), weedy (488), Rufipogon (96), wild type (245), and mutant lines (181) (**Table S3**).

The traits clum or culm length (cm), heading date, and panicle length (cm) were evaluated for GWAS, and amylose content (%), panicle length (cm), number of grains/panicle, heading date, number of panicles/hill, and 100-grain weight were used in genomic selection analysis. Phenotyping was conducted at the Kongju National University, Yesan, South Korea, during the dry seasons of 2017 and 2018. Five plants were randomly selected from the middle row of the plot, and each parameter was recorded using the Standard Evaluation System (SES) (IRRI, 2002). Days to heading were defined as the time when half of the plants in each accession showed panicles. Amylose content was determined using the iodine colorimetric method at 620 nm absorbance on a UV-1800 spectrophotometer (Shimadzu Co., Kyoto, Japan). Frequency distributions of phenotypic data were tested for normality using the Shapiro–Wilk function in R environment (Royston, 1995).

### 2.5 Genotyping

Genomic DNA was extracted from young green leaf tissue using a Qiagen plant DNeasy kit (Qiagen, Germantown, MD, USA) and quantified using a NanoDrop spectrophotometer (Thermo Scientific, USA). The DNA quality was checked using a 1% agarose gel. Genomic DNA (200 ng) from all lines was hybridized into arrays using the Affymetrix GeneTitan system, according to the manufacturer’s instructions. SNP genotyping, quality control (QC), and SNP filtering were performed according to the Axiom Genotyping Solution Data Analysis User Guide (http://www.affymetrix.com/). Briefly, genotype calling and QC metrics were performed using Affymetrix Genotyping Console™ (GTC) v.4.2. Samples with a development quality check (DQC) value<0.83 and call rate<0.97 were excluded from further analysis. GTC results were post-processed using the SNPolisher R package (v.3.0). The Ps_Metrics function was used to generate SNP metrics, and the Ps_Classification function with the default setting classified SNPs into six categories: PolyHighResolution (SNPs had good cluster resolution and at least two examples of the minor allele), MonoHighResolution (SNPs had good SNP clustering but less than two samples had the minor allele), Off-Target Variant, CallRateBelow-Threshold (SNPs had call rates CR below the threshold, but the other properties were above the threshold), NoMinorHom (SNPs had good cluster resolution but no samples had the minor allele), and Other (more than one cluster property was below the threshold) (Gao et al., 2014b). Furthermore, SNP QC metrics, including call rate (CR, ≥97%), Fisher’s linear discriminant (FLD, ≥3.6), heterozygous strength offset (HetSO, ≥_0.1), and homozygote ratio offset (HomRO, ≥0.3) values, were applied to assess SNPs. The remaining SNPs retained for further analysis were annotated using an in-house Python script. SNP distribution and count across the rice genome were analyzed using a 100 kb sliding window with an R package.

### 2.6 Genome-wide association studies

High-quality SNPs obtained from the Affymetrix chip were used in a genome-wide analysis of associations. A GWAS was performed on three phenotypic traits: clum length, heading date, and panicle length. Association analyses were conducted using the Genome Association and Prediction Integrated Tool (GAPIT) (Lipka et al., 2012) and TASSEL 5.0 (Trait Analysis by Association, Evolution, and Linkage) (Bradbury et al., 2007). GWAS analysis was performed using the mixed linear model (MLM) of GAPIT (Yu et al., 2006) to predict the association between each SNP and the phenotypic data. The kinship (K) matrix represents the variance-covariance matrix between individuals. The R package qqman (https://cran.r-project.org/web/packages/qqman/index.html) was used to draw Manhattan plots. A *P* value of 3.16 × 10^−7^ was used to consider marker-trait association (MTA) as significant.

### 2.7 GS Models

#### 2.7.1 Penalized Regression Model

The ridge regression best linear unbiased prediction (rr-BLUP) model was implemented using the R package rrBLUP (Endelman, 2011). The model is described as follows.


y=μ+Zu+ e

where *y* is an *N* × 1 vector of adjusted means for all genotypes, *μ* is the overall mean, Z is an N × M matrix of markers, u is a vector of marker effects as u ~ N(0, Iσ2u), and e is the residual error with e ~ N(0, Iσ2e).

GS was performed with fourfold cross-validation by including 80% of the samples in the training population and predicting the genomic estimated breeding values (GEBVs) of the remaining 20% of the samples. For the accuracy assessment, two 50 replication sets were performed, with each replicate consisting of five iterations.

#### 2.7.2 Convolutional Neural Networks

Convolutional neural network (CNN) is a deep learning model that accommodates inputs distributed along with space patterns (Pérez-Enciso and Zingaretti, 2019). In a CNN, the input data first passes through a convolutional layer, followed by a pooling layer, dropout layer, fully connected dense layer, batch normalization layer, and finally to the output layer containing one node with the predicted trait value. During each convolutional layer, the CNN applies kernels and filters, and performs the convolution operation with a predefined width and strides, providing the same weights for all SNP marker windows. The filter moves for the same window size across the input SNP markers, and the CNN obtains a locally weighted sum (Sandhu et al., 2021). The earlyStopping function in Keras (https://keras.io/callbacks/#earlystopping) was applied to control model overfitting (Zingaretti et al., 2020). A pooling layer is added after each convolutional layer for dimensionality reduction, and the filters are invariant to small changes in the input. Finally, pooling results in a smoothed representation and merging of the kernel output of the previous convolutional layer by taking the minimum, mean, and maximum (Bellot et al., 2018).

## 3 Result

### 3.1 Alignment, SNP selection, and Axiom Array design

A detailed description of the detection, filtering, and final selection of SNPs included in the array is provided in the Methods section and **Figure 1**. SNPs collected from eight different datasets were selected based on the breeder’s interests and key research divisions (**Figure 2**).

Alignment of whole-genome resequencing of 1) 475 Korean World Rice Collection (KRICE) accessions and 2) 3,000 rice genome project (3 K-RGP) data accessions against both indica and japonica rice reference genomes were performed to identify sequence variations (SNPs and indels) (Kawahara et al., 2013; Zhang et al., 2016). The alignment resulted in the identification of over 3.1 million SNPs in KRICE, of which 40 K high-quality SNPs (MAF< 0.01 and zero missing rates), all exonic SNPs/indels and sub-species specific SNPs were selected. Furthermore, SNPs from the high-density rice array assay from 3) Cornell University, 4) Affymetrix 44 K Rice Chip, 5) QTARO database, and 6) Plant Reactome were mapped and aligned to select potential SNPs. SNPs from (7) chloroplasts (Tong et al., 2016; Cheng et al., 2019) and mitochondrial genomes (Tong et al., 2017) of japonica and rufipogon rice were filtered and 3,449 and 3,329 SNPs/indels were selected, respectively. In GMO markers, 20,895 markers were derived from binary vectors, and 2,089, 13,697, and 746 markers derived from ds, tDNA, and tos17, respectively. As described in the ‘Methods’ section, after applying different criteria to identified SNPs data, a set of 620,852 high-quality SNPs were selected. Finally, 581,006 SNPs were tiled on the 580 K _ KNU chip SNP array that includes 3,329 from rufipogon, 34,276 from indica, and 542,432 from japonica (3,449 plastid and 538,983 nuclear). Among japonica specific 538,983 SNPs/indels (500,725 SNPs and 38,258 indels) 126,076 SNPs were for agronomic traits, 162,315 for intergenic, and 94,393 for genic SNPs (**Figure 2B**). Among the 34,276 indica-specific SNPs/indels, 22,820 were SNPs, and the remaining 11,456 were indels. In the case of 8) GMO, 969 regions were selected from transgenic genes and vectors, and a total of 40,715 probes were selected as candidate SNPs (**Figure 2B**).

**Figure 3:**
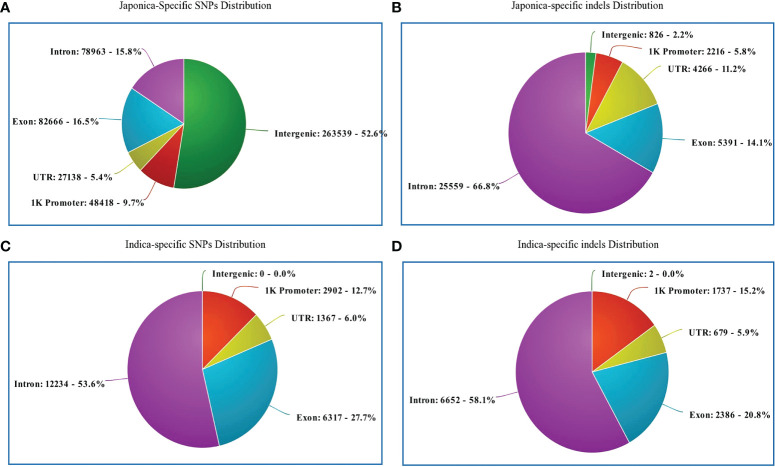
Genomic position of selected SNPs and indels. **(A)** Distribution of japonica-specific SNPs in different genomic regions. **(B)** Distribution of japonica-specific indels in different genomic regions. **(C)** Distribution of genomic regions indica-specific SNPs in different genomic regions **(D)** Distribution of indica-specific indels in different genomic regions.

**Figure 4:**
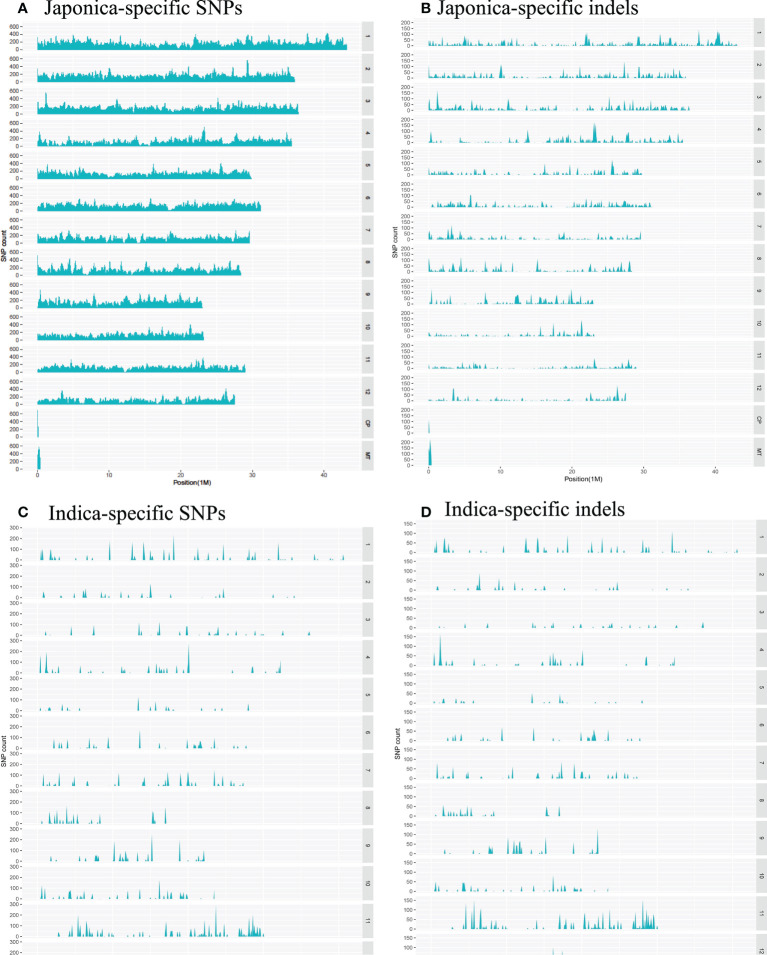
Distribution of the converted SNPs on the array in 100 Kb windows along the rice chromosomes. **(A)** Density of japonica-specific SNPs across chromosomes. **(B)** Density of japonica-specific indels across chromosomes **(C)** Density of indica-specific SNPs across chromosomes **(D)** Density of indica-specific indels across chromosomes.

Regarding the distribution of 500,725 japonica-specific SNPs in different parts of the genes, 82,666 (16.51%) SNPs were present in exons, 78,963 (15.77%) in introns, 27,139 (5.42%) in the UTR, and 26,3539 (52.63%) in the intergenic region (**Figure 3A**). Most indels were detected within an intron of japonica (**Figure 3B**). In the case of 22,820 indica-specific SNPs, no intergenic SNP were detected, whereas only two indica-specific indels were observed in the intergenic region (**Figure 3C, D**). Of the indica-specific SNPs, 53.6% (12,234), 27.7% (6,317) and 06.0% (1,367) were distributed within introns, exons, and UTR, respectively (**Figure 3C**). SNPs were located along each of the 12 rice chromosomes, with an average density of 154 SNPs/100 K and a median density of 130 SNPs/100 K (**Figure 4**). The average gap between two adjacent SNPs was 200 bp, and gaps between more than 90% were less than 2 kb (**Table S4**).

### 3.2 Genotyping performance of 580K _ KNU chip

**Figure 5:**
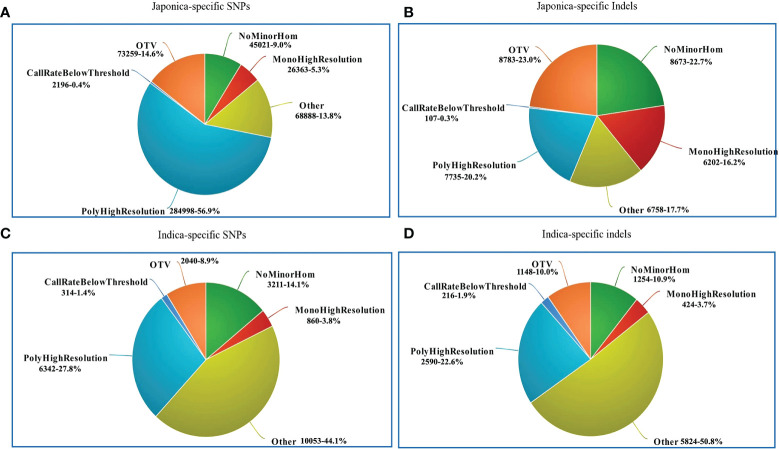
Summary of SNP genotyping data in 4720 accessions using the 580K _ KNU chip. **(A)** Classification of japonica-specific SNPs **(B)** Classification of japonica-specific indels **(C)** Classification of indica-specific SNPs **(D)** Classification of indica-specific indels. NoMinorHom: No minor homozygote, MonoHighResolution: Monomorphic high-resolution, PolyHighResolution: Polymorphic high-resolution, CallRateBelowThreshold: Call rate below threshold, OTV: Off-target variant.

The performance of the 580 K _ KNU Axiom Array was evaluated by genotyping eight different sets of genotypes on an integrated Affymetrix GeneTitan^®^ platform. All samples passed the quality assessment with a high DQC value (>0.89) and call rate (>95%), and duplicate samples showed 99% SNP reproducibility. Thus, the genotyping results of 4,720 genotypes validated the chip performance with both a high sample success rate and genotyping call. The SNP genotyping results from the 4,720 genotypes were classified into six categories based on the Affymetrix quality control metrics (**Table 1**; **Figure 5**). Based on the filtering parameters, Fisher’s linear discriminant (FLD), HetSO, HomRO, and CR ≥95% as the filtering options, approximately 79.2% (n = 18,087) of the total indica-specific array SNPs were converted. Of the 38,258 designed japonica-specific indels, 23,759 passed the bead representation and decoding quality metrics. In japonica-specific SNPs, 53.13% SNPs were categorized in the ‘PolyHighResolution’ category, whereas 15.94% SNPs were found in ‘OTV’ and 6.08% in the ‘MonoHighResolution’ category (**Figure 5A**). A total of 7,735 japonica-specific indels (20.2%) were classified ‘PolyHighResolution’ and 23.0% as ‘OTV’ (**Figure 5B**). While in the case of indica-specific SNPs, 24.8% of SNPs were found in the ‘PolyHighResolution’ category, but the highest SNPs (42.6%) were grouped fall in the ‘other’ category (**Figure 5C**). A similar trend was found for indica-specific indels with the highest 50.8% in ‘other’ category, followed by 22.6% of SNPs in the ‘PolyHighResolution’ (**Figure 5D**).

**Table 1:** Classification of SNPs in the 580K SNP Chip after genotyping on 4720 accessions.

SNP category	Japonica-specific SNPs	Japonica-specific indels	Indica-specific SNPs	Indica-specific indels
NoMinorHom	45,021	8,673	3,211	1,254
MonoHighResolution	26,363	6,202	860	424
PolyHighResolution	284,998	7,735	6,342	2,590
CallRateBelowThreshold	2,196	107	314	216
OTV	73,259	8,783	2,040	1,148
Other	68,888	6,758	10,053	5,824
Total	500,725	38,258	22,820	11,456

NoMinorHom: No minor homozygote, MonoHighResolution: Monomorphic high-resolution,

PolyHighResolution: Polymorphic high-resolution, CallRateBelowThreshold: Call rate below threshold, OTV: Off-target variant.

### 3.3 Genome-wide association studies and genomic selection

**Figure 6:**
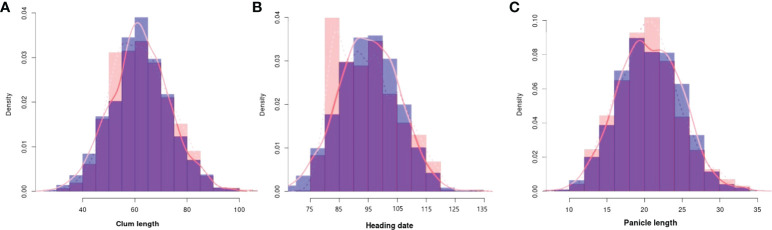
Frequency distribution for all the traits used for the genome-wide association study. **(A)** Clum length, **(B)** Heading date, and **(C)** Panicle length. The histograms with purple colors are normal as expected while other colors denote difference from normal distribution. The observed and expected normal distributions fitting for data were represented with dashed and solid red lines, respectively.

**Figure 7:**
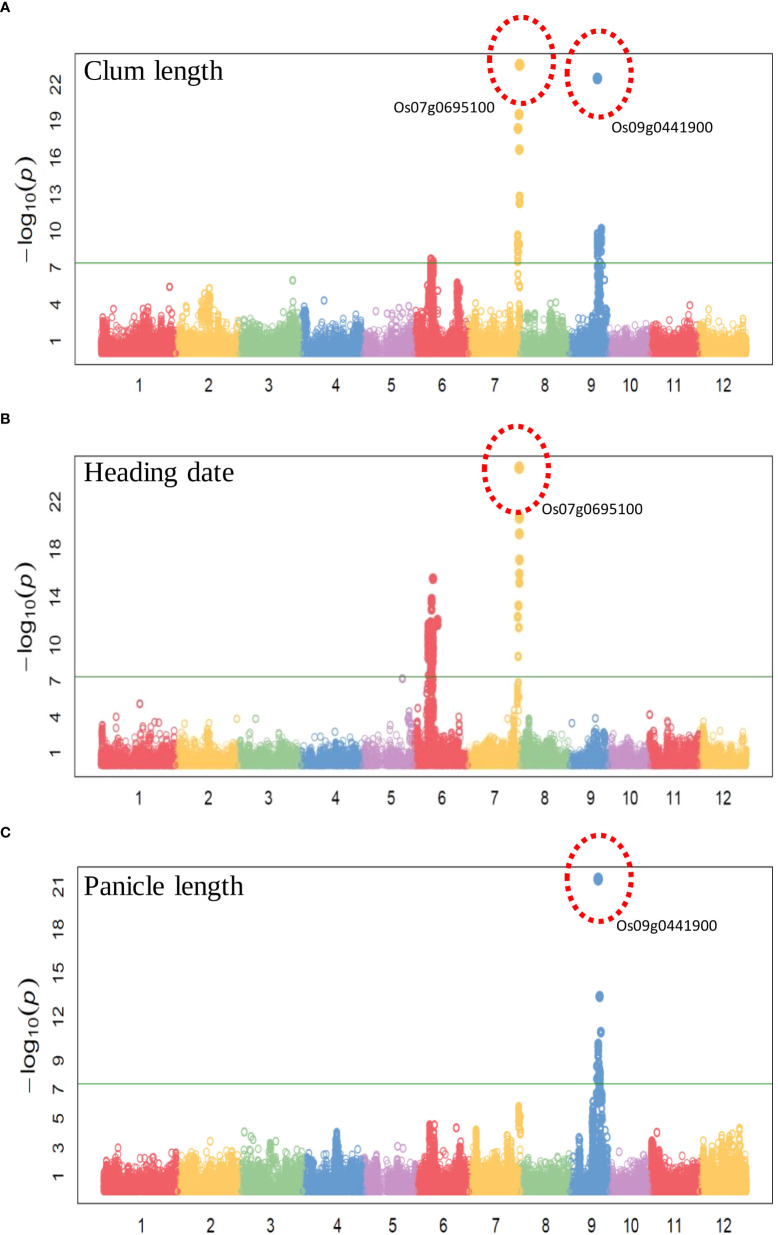
Manhattan plots of genome-wide association studies using the 580K _ KNU chip **(A)** Manhattan plot for Clum length, **(B)** Manhattan plot for Heading date, and **(C)** Manhattan plot for Panicle length.

To evaluate the practicality of this array in GWAS, we used a set of 1,288 lines, representing a subset of the 4,720 collection that were phenotyped for clum length, heading date, and panicle length. The frequency distribution curves revealed continuous variation and displayed a normal distribution for all the traits (**Figure 6**). A total of 60 SNPs were detected for clum length with a threshold above 7 (−log_10_ *p*-value) having the highest −log_10_ *p*-value of 22 for a marker on chromosome 7 (chr07_ 29494004) (**Figure 7A**; **Table S5**). In contrast, 277 markers on chromosomes 6 and 7 were significantly associated with heading date with a 7< −log_10_ *p*-value (**Figure 7B**; **Table S6**). We obtained a total of 84 significant SNPs exceeding the threshold –log_10_ *p-*values of 7 for panicle length (**Figure 7C**; **Table S7**). From the GWAS results, we observed several significantly associated SNPs with three traits located on chromosomes 6, 7, and 9 based on the genome-wide significance cut-off, −log_10_ *p-*values 7 (**Figure 7**). For panicle length, three SNPs from *Os09g0456100* (OsLP1; LONG PANICLE 1) showed significant association with −log_10_ *p*-value of 7<. In addition, significant SNPs mapped on the 2.4 Mbp region of chromosome 9 were belongs to 15 genes that include *DENSE AND ERECT PANICLE1* (*DEP1; Os09g0441900*), *lecithine cholesterol acyltransferase (Os09g0444200), Serine carboxypeptidase 42 (Os09g0462875)*, gibberellin receptor (*Os09g0455900*), alpha-amylase isozyme 3A precursor (*Os09g0457400*), etc were significantly associated with panicle length (**Table 2**). Similarly, annotation of significant SNP regions with -log_10_ *p-*values > 7 predicted a total of 42 genes on chromosomes 6 and 7 for heading date and 12 genes for clum length (**Tables S8**).

**Table 2:** List of genes identified from significantly associated SNPs for phenotypic traits.

Trait	SNP ID	Chr	Position	SNP effect	−Log10 (P)	Locus_ID	Description
Clum length	AX-281898346	7	29623752	6.79	23.60	*Os07g0695100*	OsPRR37; Heading date 2, PSEUDO-RESPONSE REGULATOR 37
	AX-95959478	9	16415391	4.95	22.51	*Os09g0441900*	OsDEP1; DENSE AND ERECT PANICLE 1
	AX-115865280	7	29529536	-2.74	12.32	*Os07g0693700*	WD40 repeat-like domain-containing protein.
	AX-154994951	9	18671171	2.90	9.23	*Os09g0483400*	Similar to Ubiquitin/ribosomal fusion protein (Fragment).
	AX-117368794	9	16535714	-4.27	8.98	*Os09g0444100*	Similar to Minus dominance protein.
	AX-95931544	9	17878923	3.07	7.06	*Os09g0469900*	Similar to Queuine tRNA-ribosyltransferase.
	AX-95938232	9	16543763	-5.44	7.04	*Os09g0444200*	Lecithin: cholesterol acyltransferase family protein.
Heading date	AX-115865280	7	29529536	-3.34	20.65	*Os07g0693700*	WD40 repeat-like domain-containing protein.
	AX-281898346	7	29623752	5.75	17.17	*Os07g0695100*	OsPRR37; Heading date 2, PSEUDO-RESPONSE REGULATOR 37
	AX-115788386	7	29623803	3.89	15.96	*Os07g0695100*	OsPRR37; Heading date 2, PSEUDO-RESPONSE REGULATOR 37
	AX-283557343	7	29632090	3.78	15.26	*Os07g0695300*	OsRLCK243: Receptor-like Cytoplasmic Kinase 243
	AX-117367125	6	10148113	-4.49	11.19	*Os06g0285400*	Similar to Serine/threonine-specific kinase-like protein.
	AX-154504166	6	10588394	3.99	10.86	*Os06g0289900*	UDP-glucuronosyl/UDP-glucosyltransferase family protein.
	AX-117355228	6	10377892	-4.46	10.66	*Os06g0286500*	Similar to NBS-LRR disease resistance protein homolog.
	AX-123161598	6	10347685	-4.45	10.66	*Os06g0286351*	Armadillo-type fold domain-containing protein
	AX-155742676	6	10480694	4.18	10.29	*Os06g0288300*	OsCGT; C-glucosyltransferase, Flavone-C-glycoside synthesis
	AX-155780256	6	10343235	4.04	10.27	*Os06g0286310*	Similar to Oxidoreductase-like protein.
	AX-116841734	6	8197078	3.94	9.98	*Os06g0257200*	Similar to Signal recognition particle 9 kDa protein.
	AX-154042176	6	10959896	4.02	9.77	*Os06g0296700*	C-type lectin, conserved site domain-containing protein.
	AX-154664097	6	8313556	3.80	9.54	*Os06g0258900*	NAD(P)-binding domain-containing protein.
	AX-155544336	6	10553998	3.96	9.47	*Os06g0289200*	UDP-glucuronosyl/UDP-glucosyltransferase family protein.
	AX-273967585	6	9319713	-2.58	9.34	*Os06g0274500*	OsSERL2; Somatic embryogenesis receptor kinase-like 2
	AX-117351923	6	10438340	3.82	8.75	*Os06g0287500*	CC-NBS-LRR protein, Blast resistance
	AX-154759957	6	10374435	3.55	7.87	*Os06g0286375*	Similar to Nitrate-induced NOI protein-like protein.
	AX-117364893	6	10131338	-3.16	7.79	*Os06g0285100*	*OsFLA23*; fasciclin-like arabinogalactan protein 23
	AX-117360522	6	10184468	-3.39	7.40	*Os06g0285900*	Similar to embryogenesis transmembrane protein.
Panicle length	AX-95959478	9	16415391	1.91	21.49	*Os09g0441900*	*OsDEP1*; DENSE AND ERECT PANICLE 1
	AX-95938232	9	16543763	-2.12	9.65	*Os09g0444200*	Lecithin: cholesterol acyltransferase family protein.
	AX-283693666	9	15591135	-1.35	8.62	*Os09g0429000*	*OsGLR2.1*; Glutamate receptor homolog 2.1.
	AX-115821718	9	17185419	-1.88	8.46	*Os09g0456100*	*OsLP1*; LONG PANICLE 1.
	AX-117360521	9	17109742	-1.91	8.39	*Os09g0455200*	*OsHSF22*; Heat stress transcription factor 22.
	AX-117388485	9	17290563	-1.91	8.23	*Os09g0457400*	*OsEnS-129*; Alpha-amylase3A.
	AX-155718545	9	17506122	-1.82	8.01	*Os09g0462875*	*OsSCP42*; Serine carboxypeptidase 42.
	AX-276235752	9	17081620	-1.86	7.90	*Os09g0454600*	*OsPT19*; Phosphate transporter 19
	AX-116843575	9	17185192	-1.81	7.43	*Os09g0456100*	*OsLP1*; LONG PANICLE 1.
	AX-154875030	9	17172676	-1.71	7.39	*Os09g0455900*	Alpha/beta hydrolase fold-3 domain-containing protein.
	AX-117388873	9	17184182	-1.77	7.23	*Os09g0456100*	*OsLP1*; LONG PANICLE 1.

**Figure 8:**
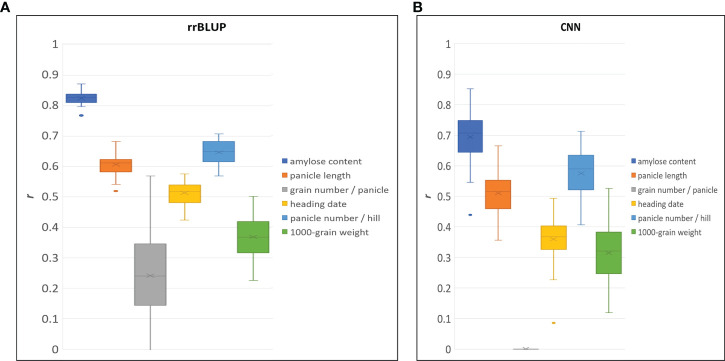
Genomic selection predictive ability (r) for different traits by using rrBLUP **(A)**, and CNN **(B)** model.

To demonstrate the performance of the 580 K _KNU chip rice array in quantitative phenotype prediction, we utilized the rrBLUP and CNN statistical models. The predictability of genomic selection was evaluated using four-fold cross-validation, where the sample was randomly partitioned into four parts to estimate the parameters. Finally, all parts were predicted once and used ten times to estimate the parameters. A total of 80, 7 genotypes, including having bred and weedy rice and corresponding 121,208 markers from chip data, were used to evaluate the accuracy of prediction for amylose content (**Table S9**). Both models showed the highest predictability for amylose content, and values of predictability for all traits with rrBLUP were higher than those of CNN (**Figure 8**). The predictabilities of the three traits were more than 0.5 in both models. The CNN model was unable to predict the grain number per panicle; however, rrBLUP had a lower value (**Figure 8**).

## 4 Discussion

Rice was the first crop species to be fully sequenced (International Rice Genome Sequencing Project, 2005). Thousands of varieties have been re-sequenced (The 3,000 rice genomes project, 2014; Duitama et al., 2015), and *de novo* assemblies have been performed for several subspecies (Yu et al., 2002; Kawahara et al., 2013; Schatz et al., 2014). Based on accrued genomic information, QTL studies and GWAS have been good strategies for understanding the genetic basis underlying complex traits in rice (Yano et al., 2019; Wang et al., 2020; Yuan et al., 2020), and major associations have been identified as important traits for the last few decades. In current rice breeding programs, a major limitation in the genetic dissection of agronomically important traits is the tight linkage between undesirable loci and preferable loci, for example, two loci within a linkage disequilibrium (LD) block (Xiao et al., 2021). To break tightly linked loci, breeding experts either have to enlarge the population size or advance generations until the block is dissected and the marker interval is minimized. High-throughput genotyping arrays can genotype hundreds of thousands of markers over a large number of samples in a short timeframe. Owing to this technology, the breeding system enables the handling of the population required to dissect LD blocks in a relatively short time. Through association studies and linkage mapping, the rate of development of trait-linked DNA markers can be accelerated, and the breeding cycle can be dramatically reduced, even for tightly linked traits within LD blocks. In the current rice breeding program, where major associations have been detected for important traits, SNP array chips must be designed based on breeders’ interests to address challenging problems in rice breeding, along with efficient and fast genotyping.

In this study, the 580 K _KNU chip array was developed based on inclusive genomic polymorphisms targeting breeders’ interests, covering all key traits and research areas. To construct an integrated array system, in addition to the SNPs from 475 KRICE, SNPs from major rice chips that had been published previously were added after strict filtration (**Figure 1**). To make the 580 K _KNU chip more informative for the current rice breeding program, we selected candidate SNPs associated with important traits to reflect breeders’ interests (**Figure 2**). Hence, along with a dense marker interval of 200 bp, the 580 K _KNU chip consisting of 581,006 high-quality SNPs representing the genomic polymorphisms in rice is an excellent platform for genetic dissection of agronomic traits and is highly informative, especially for current research topics in rice breeding programs. The development and cultivation of GM crops have expanded over time, and microarray technology is a flexible method of detecting GMO varieties. In this study, we incorporated GMO-specific loci covering 40,715 markers that will be helpful in rice breeding for preliminary screening of GMOs contaminations. Previously such microarray-based GMO detection has been applied at a small scale in detection of GM soya, rice and maize lines (Kim et al., 2010; Turkec et al., 2016; Chen et al., 2021; Kutateladze et al., 2021).

Using the 580 K _KNU chip, we evaluated the associations between the three phenotypes and the 580 K SNP genotypes in different lines. For club length and heading date, the most significant signals were located in the candidate gene *Os07g0695100* (Pseudo-Response Regulator37; *OsPRR37*), which was identified as being responsible for the early heading7-2 (EH7-2)/heading date2 (Hd2) QTL (Koo et al., 2013; Yan et al., 2013; Gao et al., 2014a). In the case of panicle length, the most significant signal (chr09_16415391 with −log10 *p*-value 21) was observed in the *OsDEP1* gene (*Os09g0441900*) (**Table 2**), which controls the erect panicle (EP) architecture, which is a typical characteristic of super rice utilized in rice breeding for nearly a century owing to its high yield, lodging tolerance with strong stems, reasonable population structure, and high nitrogen use efficiency (Huang et al., 2009; Zhou et al., 2009; Xu et al., 2016; Sibo et al., 2021). The same marker chr09_16415391 (*Os09g0441900*) was found to be the second most significant signal (−log10 *p*-value 22) for clum length (**Table 2**). *Os09g0456100* (OsLP1; LONG PANICLE 1) that encodes Remorin_C-containing proteins showed significant association with panicle length. Previously, two SNPs from the third and fifth exons of *LP1* were reported to reduce panicle length (Liu et al., 2016). The clusters of significant SNPs associated with heading date were detected around *Os07g0695100* covering an extensive region of 0.15 Mb, followed by a region (~1-0.89 Mb) on chromosome 6 containing *Os06g0285900* (embryogenesis transmembrane protein), *Os06g0285100* (*OsFLA23*; fasciclin domain-containing protein) *Os06g0286310* (Oxidoreductase-like protein), *Os06g0286400* (nitrate induced protein), *Os06g0289200* (UDP-glucuronosyl/UDP-glucosyltransferase family protein), *Os06g0286351* (Armadillo-type fold domain containing protein), *Os06g0285400* (Serine/threonine-specific kinase-like protein), *Os06g0296700* (C-type lectin conserved site domain-containing protein) which have not previously been shown to be associated with any trait in rice (**Table 2**). Similarly, for heading date, 34 and 13 QTLs were detected near *Hd1* and *Hd17*, *RFT1*, and *Hd3a* genes, respectively, on chromosome 6, while 10 QTLs were detected near the *OsPRR37* gene on chromosome 7 (Ebana et al., 2011; Hori et al., 2015). Therefore, identified candidate loci could be targeted for fine mapping in order to determine the exact genes/alleles underlying these GWAS signals pertaining to agronomic traits.

GBLUP is the most robust method and the most commonly used tool in rice because it provides high predictability (Xu et al., 2014; Spindel et al., 2015; Xu et al., 2021). In GS selection using the 580K_KNU chip, rrBLUP performed better than CNN, but both models showed > 0.5 prediction values (**Figure 8**). GS predictability is influenced by various factors, including heritability, relatedness between populations, sample size, marker density, genetic architecture, statistical model, and factors. GS accuracy in rice breeding populations has been performed for various quantitative traits varied by trait, population, and model and moderate to high predictive ability has been reported (Xu et al., 2021). Onogi et al., 2015 recorded the predictive ability of heading date (0.8), clum length (0.75), panicle length (0.6), panicle number (0.4), and grain length (0.4) in a population of 110 Asian rice cultivars using GBLUP (Onogi et al., 2015). The genotyping of 413 rice inbred lines with a 44 K chip showed the predictive abilities for florets per panicle (~0.6), flowering time (~0.63), plant height (~0.7), and protein content ~0.44 (Isidro et al., 2015), while in a panel of 363 elite breeding lines predictive abilities of 0.31, 0.34, and 0.63 for grain yield, plant height and flowering time, respectively were reported (Spindel et al., 2015). The predictive abilities for 1000 grain weight were 0.82–0.83 in 210 recombinant inbred lines and 278 hybrids (Xu et al., 2014), and 0.54 in 1495 hybrids derived from incomplete NC II design (Cui et al., 2020). The GS predictive values reported in this study were in accordance with previous reports on rice (Xu et al., 2014; Grenier et al., 2015; Spindel et al., 2015; Xu et al., 2018; Cui et al., 2020). Compared with other arrays, where several major genes have not been fully integrated, our 580 K _KNU chip system might be better suited for GS. Similarly, the gene–coding sequence–haplotype (gcHap)-based GS showed higher predictive ability than SNP-based GS because the gcHap dataset represents the diversity of 45 963 rice genes in 3010 rice accessions (Zhang et al., 2021). GS-specific SNP arrays could further improve rice breeding accuracy, intensity, and efficiency as well as reduce cost and time.

The current manuscript mainly focused on the development of an inclusive SNP array system that will help rice breeders for multiple breeding programs. Also, we have conducted validation analyses for the GWAS and genomic selection. In collaboration with DNA Link, Inc. (Korea), we provide microarray analysis services using our 580 K _KNU chip. Scientists and breeders in Korea are using it for a variety of applications, and we expect to see the results of studies on the use of our SNP array in the near future. As an accurate high-density genotyping tool, the 580 K _KNU chip is an excellent platform for GWAS, QTL mapping, evolutionary studies, genetic diversity, and genomic selection, especially genetic dissection of important traits of breeders’ interest that have not been fully identified. Hence, it will play a pivotal role in rice breeding applications.

## Data availability statement

The original contributions presented in the study are included in the article/**Supplementary Material**, further inquiries can be directed to the corresponding author/s.

## Author contributions

K-WK, BN and JH wrote the manuscript. K-WK, JN, BN and S-HC collected and analyzed the data. JH and Y-JP supervised and revised the manuscript. All authors contributed to the article and approved the submitted version.

## Funding

This study was supported by a National Research Foundation of Korea (NRF) grant funded by the Korean government (MSIT) (No. 2022R1A4A1030348). This work was supported by the Korea Institute of Planning and Evaluation for Technology in Food, Agriculture and Forestry (IPET) through the Digital Breeding Transformation Technology Development Program funded by the Ministry of Agriculture, Food and Rural Affairs (MAFRA) (322060031HD020). This work was carried out with the support of “Cooperative Research Program for Agriculture Science and Technology Development (Project No. PJ015935), Rural Development Administration, Republic of Korea. This work was supported by a research grant from the Kongju National University in 2022.

## Conflict of interest

The authors declare that the research was conducted in the absence of any commercial or financial relationships that could be construed as a potential conflict of interest.

## Publisher’s note

All claims expressed in this article are solely those of the authors and do not necessarily represent those of their affiliated organizations, or those of the publisher, the editors and the reviewers. Any product that may be evaluated in this article, or claim that may be made by its manufacturer, is not guaranteed or endorsed by the publisher.
